# Bioanalytical Method Development Using Liquid Chromatography with Amperometric Detection for the Pharmacokinetic Evaluation of Forsythiaside in Rats

**DOI:** 10.3390/molecules21101384

**Published:** 2016-10-16

**Authors:** Yu-Tse Wu, Meng-Ting Cai, Chih-Wei Chang, Ching-Chi Yen, Mei-Chich Hsu

**Affiliations:** 1 School of Pharmacy, Kaohsiung Medical University, Kaohsiung 807, Taiwan; cappricio2012@gmail.com (M.-T.C.); wxes9050304@gmail.com (C.-W.C.); date0315@hotmail.com (C.-C.Y.); 2 Department of Sports Medicine, Kaohsiung Medical University, Kaohsiung 807, Taiwan; meichich@kmu.edu.tw

**Keywords:** forsythiaside, amperometric detection, pharmacokinetics, thermoresponsive hydrogel

## Abstract

An analytical method entailing high-performance liquid chromatography coupled with electrochemical detection was developed for determining forsythiaside (FTS) in rat plasma. Rat plasma samples were prepared through efficient trichloroacetic acid deproteination. FTS and the internal standard were chromatographically separated on a reversed-phase core-shell silica C18 column (100 mm × 2.1 mm, i.d. 2.6 μm), with a mobile phase consisting of an acetonitrile—0.05-M phosphate solution (11.8:88.2, *v*/*v*), at a flow rate of 400 μL/min. The calibration curve, with *r*^2^ > 0.999, was linear in the 20–1000 ng/mL range. The intra- and interday precision were less than 9.0%, and the accuracy ranged from 94.5% to 106.5% for FTS. The results indicated that the newly developed HPLC-EC method is more sensitive than previous reported methods using UV detection, and this new analytical method is applied successfully for the pharmacokinetic study of FTS. The hydrogel delivery system can efficiently improve bioavailability and mean residual time for FTS, as evidenced by the 2.5- and 6.3-fold increase of the area under the curve and the extension of the half-life, respectively.

## 1. Introduction

**Figure 1:**
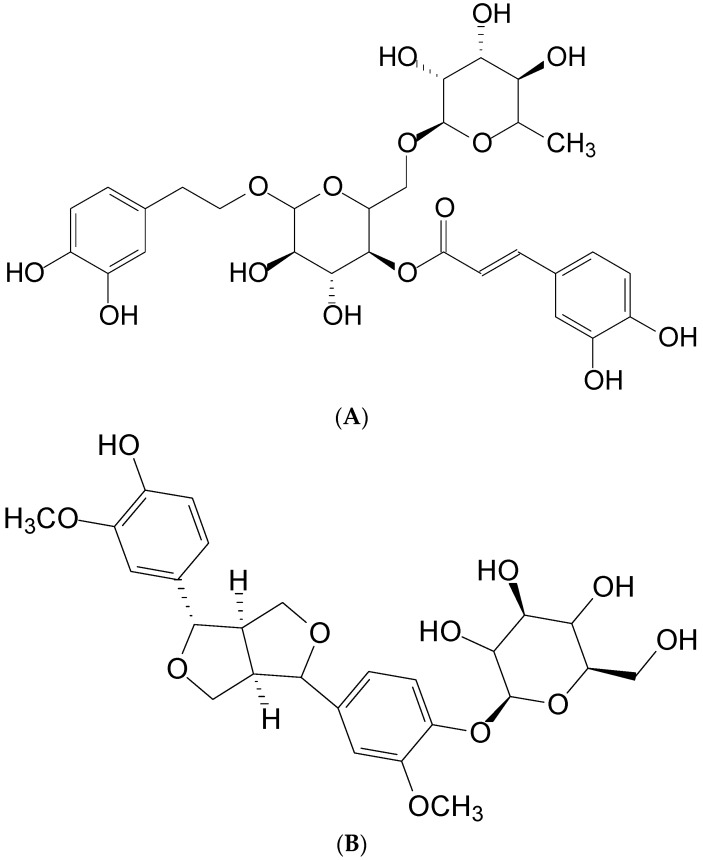
Chemical structure of (**A**) forsythiaside and (**B**) pinoresinol as the internal standard.

Forsythiaside (FTS, Figure 1), a phenylethanoid glycoside, is a major ingredient isolated from Fructus Forsythiae [1]. This herb, called Lian-Qiao in traditional Chinese medicine (TCM), has been widely used to treat different infectious diseases, such as upper respiratory tract complaints caused by bacteria and viruses [2]. Shuang-Huang-Lian powder injection, a preparation containing extracts from Fructus Forsythiae is indicated to treat infectious diseases in TCM [3]. FTS has been used for preserving neuronal structure and function through the reduction of oxidative stress, a major strategy to treat Alzheimer disease and dementia [4]. In addition, FTS has been proposed as a possible treatment for chronic inflammatory conditions caused by the influenza A virus by suppressing the release of chemokine (C-C motif) ligand 5 (also known as RANTES) from human bronchial epithelial cells [5]. FTS also possesses antibacterial activity against *Escherichia coli*, *Pseudomonas aeruginosa*, and S*taphylococcus aureus*. The minimal inhibitory concentration of FTS for *S. aureus* is even lower than that for tetracycline [6].

The pharmacokinetic evaluation of herbs is imperative because studies involving laboratory animals will provide useful information for the development of TCM, which in turn would facilitate the further evaluation of preclinical toxicological results of TCM for extrapolation to humans. In addition, during the early screening stages, evaluations of pharmacokinetics and bioavailability of potential lead compounds would become increasingly crucial if the main functional assays are molecule-based approaches, such as isolated protein assays or phenotypic assays using cells or model organisms [7]. Analytical methods with better sensitivity can collect more comprehensive time—concentration results and thus enhance the validity of the pharmacokinetic data [8]. Several pharmacokinetic studies of FTS have utilized high-performance liquid chromatography with ultraviolet detection (HPLC-UV) for analyzing rat and beagle dog plasma samples [1,9,10,11,12,13]. Similarly, HPLC with mass spectrometry (HPLC-MS) has been proposed for the pharmacokinetic evaluations of FTS [2,14,15,16,17]. HPLC-MS provides excellent selectivity and sensitivity but susceptible to matrix effects, that is, instable ionization efficiency caused by the competition between analytes and co-eluted components from the sample matrices, including phospholipids in plasma and additives to the mobile phases [18]. HPLC coupled with electrochemical detection (HPLC-EC) is an attractive alternative for determining FTS because it provides higher selectivity and sensitivity than does HPLC-UV and because it is not susceptible to matrix effects [19]. Pharmacokinetic studies have demonstrated that the half-life (t_1/2_) of FTS after intravenous administration ranges from 23.6 to 72.6 min in rats [13] and 64.3 to 89.4 min in beagle dogs [9]. The elimination t_1/2_ of protein unbound FTS was measured to be 12.7 ± 3.4 and 18.2 ± 5.5 min for rat blood and bile, respectively [1]. The therapeutic effectiveness and outcomes usually depend on whether the appropriate drug concentrations are achieved. In addition, therapeutic agents with short a half-life will need frequent administration to maintain the therapeutic effects.

FTS possesses antimicrobials effects against infectious diseases, but the major hindrances to its clinical applicability are its low oral bioavailability (approximately 0.5%) and rapid elimination, which may result in insufficient drug concentration in systemic blood circulation after drug administration. To prolong the t_1/2_ of drugs, the in situ forming depot formulations are an ideal choice instead of frequent administration or constant infusion [20]. The in situ formation depot formulations has been proposed and investigated for controlled drug delivery in systemic treatments and localized therapies, and this formulation remains aqueous with a low viscosity but turns into a semisolid or solid depot after administration. The thermally-induced gelling hydrogel system is a type of in situ forming depot formulations, and is considered an intelligent drug-delivery system because it transitions from solution to gel conditions in response to an increase in the surrounding temperature [21]. Hydrogel drug-delivery systems are multicomponent systems consisting of a three-dimensional network of polymer chains and water molecules that fill the space between the macromolecules [22]. With such structural characteristics, hydrogels can sense the surrounding stimuli and respond by altering their chemical or physical status, leading to the controlled release of embedded active ingredients. In this study, we developed and validated a HPLC-EC method with an efficient protein precipitation pretreatment to determine FTS concentrations in rat plasma. The method was further applied to the in vivo pharmacokinetic characterization of FTS. Further, we designed an extended delivery formulation utilizing thermally-induced gelling hydrogel to solve the relatively short in vivo t_1/2_ for FTS. The FTS-loaded hydrogel formulations were optimized according to the sol-gel transition temperature (T_sol-gel_), temperature-viscosity profiles, and gel dissolution-drug release correlations.

## 2. Results

### 2.1. Bioanalytical Method Validation for FTS

#### 2.1.1. Selectivity

**Figure 2:**
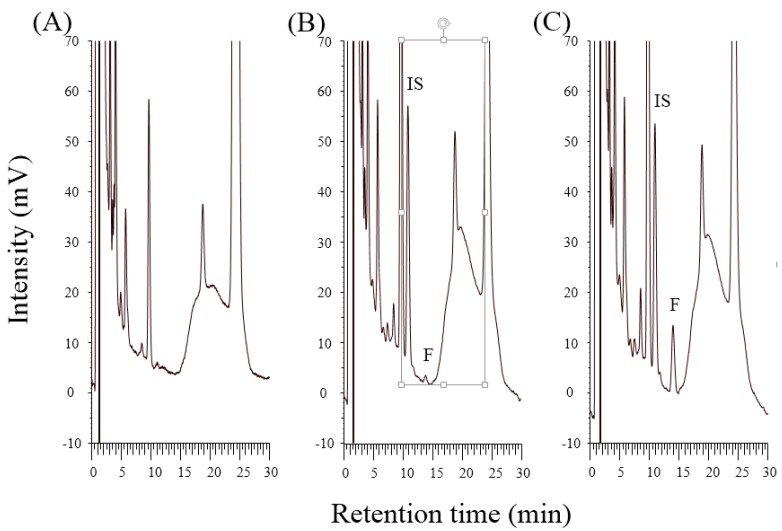
HPLC-EC chromatograms of (**A**) blank rat plasma; (**B**) blank plasma spiked forsythiaside (20 ng/mL) and (**C**) plasma sample collected 15 min after subcutaneous injection of forsythiaside hydrogel (3 mg/kg).

Analytical method selectivity was examined by analyzing blank plasma for six different rats. No obvious interference peaks appeared around the retention times of FTS (13.6 min) and IS (10.7 min), as shown in the typical chromatograms (Figure 2). FTS is a caffeoyl glycoside consists of caffeic acid, 3,4-dihydroxy-β-phenethyl alcohol, d-glucose and l-rhamnose. The two genins and the two sugar moieties are polar and less retained on the reversed-phase stationary phase, and should appear before FTS in the chromatogram. The validation results indicated that the current method was reproducible and reliable. Therefore, the interference caused by metabolites of FTS is minimal and can be ignored.

#### 2.1.2. Linearity and Sensitivity

The linear regression equation of FTS was y = 0.0009x − 0.0031, and all *r*^2^ values were greater than 0.999, which confirmed good linearity over the concentration range (20–1000 ng/mL). The limit of detection (LOQ) and (LOD) for FTS were 20 and 10 ng/mL, respectively.

#### 2.1.3. Recovery

Recovery (*n* = 3) for FTS was found to be 86.2% ± 5.1%, 83.4% ± 2.0% and 79.5% ± 3.6% for low quality control (LQC: 40 ng/mL), middle quality control (MQC: 400 ng/mL), and high quality control (HQC: 750 ng/mL) samples, respectively. The recovery for ISTD was 82.1% ± 5.7%.

#### 2.1.4. Accuracy and Precision

The intraday accuracy of FTS ranged between 94.5% and 106.5%, and the inter-day accuracy ranged between 96.6% and 99.8% (Table 1). The intra- and inter-day precisions, expressed as RSD, for FTS in rat plasma samples were found to be less than 7.5% and 9.0%, respectively (Table 1), which confirmed the high precision of the developed method.

#### 2.1.5. Stability and Dilution Integrity

Methanolic stock solutions of FTS were found to remain stable for 3 months at −70 °C, with the mean stability ranging from 95.8% to 106.6%. As shown in Table 2, the accuracy for FTS and ISTD in the plasma samples was within 15%, which is acceptable and can be considered stable for 24 h within the autosampler [23]. Table 3 listed the stability results of benchtop and freeze-thaw tests. We have also evaluated the dilution integrity, and the results in Table 4, which indicated that the dilution steps have ignorable effects on the precision and accuracy.

### 2.2. Characterization of Hydrogel Formulations

The temperature-viscosity and gel dissolution profiles of the seven formulations are shown in Figure S1. The T_sol-gel_, gelling time, maximum viscosity, and complete dissolution time of the formulations are presented in Table 5. Formulation 1 (F1), simply containing F127, exhibited a T_sol-gel_ of approximately 26 °C. The additions of F68 and PEG 6000 both clearly increased T_sol-gel_ from 28 °C to 38 °C. F2 fortified with 1% (*w*/*w*) F68 had a gelling time of 1.4 min, and F5 containing 0.5% (*w*/*w*) PEG 6000 had a gelling time of 1.7 min. The increasing percentages of PEG 6000 in formulations decreased the maximum viscosities as reflected in F5 to F7, whereas F68 had limited effects on the maximum viscosities. F2 and F3 achieved complete dissolution around 96 h whereas other formulations needed 72 h or fewer.

**Figure 3:**
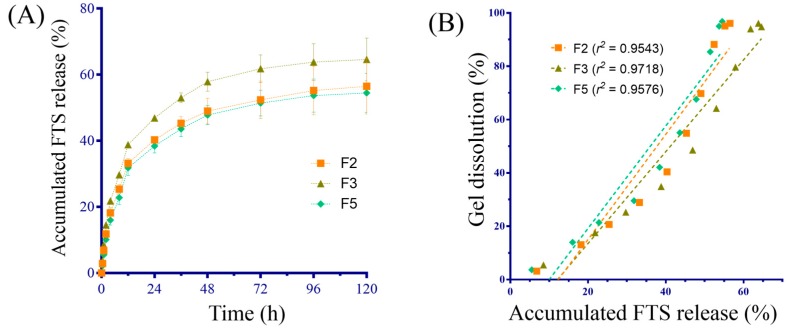
(**A**) The accumulative drug release rate of F2, F3 and F5 (*n* = 3); (**B**) Correlation profile of the accumulative percent of FTS released and the accumulative percent of thermo-sensitive hydrogel dissolved (*n* = 3 for each formulation).

A scoring criterion (Table S1) for the performance of hydrogel was developed to objectively judge the blank hydrogel formulations. By considering the importance of each physicochemical property such as T_sol-gel_, gelling time, maximum viscosity, and complete dissolution time, F2, F5, and F3 were selected for further FTS release evaluation. The accumulated FTS release profiles reached around 60% as shown in Figure 3A, and no difference was found among the three formulations (Figure 3A). The correlation coefficients between FTS release and gel dissolution were higher than 0.95 as shown in Figure 3B, indicating that the release was mostly controlled by the dissolution of the hydrogel [24]. The release kinetics results of FTS-loaded hydrogels (Table S2) suggested that the Higuchi model (*r*^2^ = 0.9114~0.9223) was more appropriate than were the zero-order model (*r*^2^ = 0.7153–0.7351) and the first-order kinetic model (*r*^2^ = 0.4066–0.4422).

### 2.3. Pharmacokinetic Evaluation of FTS

**Figure 4:**
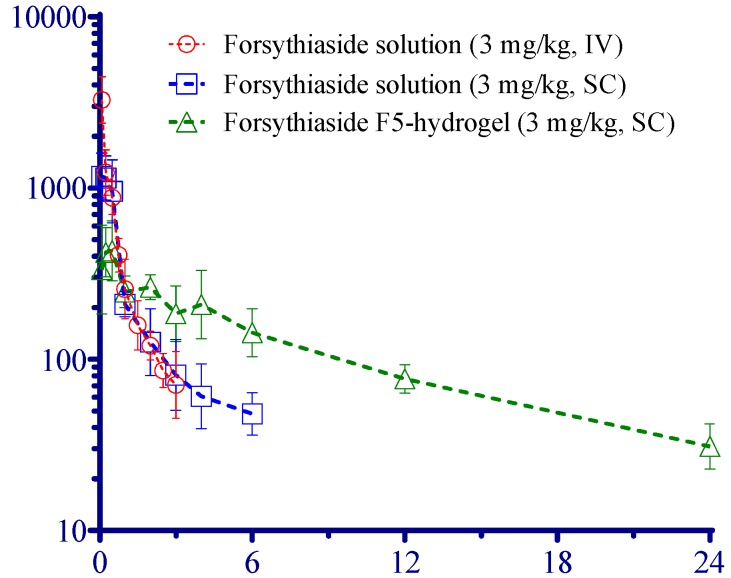
Concentration of forsythiaside in rat plasma vs. time curve after administration of drug (3 mg/kg, *n* = 4 for each group).

The results of pharmacokinetic studies are listed in Figure 4 and Table 6. The t_1/2_ of subcutaneous (SC) FTS-loaded hydrogel (516.6 min) was significantly longer compared with the t_1/2_ for free FTS (76.6 min). In addition, the clearance (Cl) of FTS-loaded hydrogel was 16.5 mL·kg/min, which is significantly lower than that of free FTS (38.5 mL·kg/min). Finally, the hydrogel delivery system exhibited higher FTS bioavailability, as indicated by the AUC (161396 vs. 74894 min·ng/mL).

## 3. Discussion

Botanic polyphenols possess diverse health-promoting benefits and their pharmacokinetic information has recently drawn much attention. Pharmacokinetic data can provide guidance for the safe and rational use of botanic and food supplements [25]. Analytical methods with high sensitivity facilitate the collection of more complete time-concentration profiles and improve the validity of the pharmacokinetic data. Table 7 listed liquid chromatographic methods of FTS for analytical performance comparison. EC is an appropriate choice for determining FTS concentrations, because EC provides higher selectivity and sensitivity than does UV; moreover, EC is not as susceptible to matrix effects as is MS. The potentials applied to the working electrode of EC were optimized to obtain the appropriate sensitivity for FTS. Higher potentials generally led to increased sensitivity as well as baseline noise and much longer equilibrium times. We therefore selected +900 mV to ensure adequate sensitivity while maintaining stable performance.

FTS is a phenylethanoid glycoside with polar properties (Log *p* = −0.5), which addresses the challenge of analysis in aqueous biological matrices [27]. To obtain adequate separation from the endogenous interference peaks, the composition of the mobile phase and the sample preparation methods were evaluated. Adjusting the pH (2.5, 3.0, 3.5, and 4.0) of the mobile phase improved the FTS peak shape as a pH > 3.5 resulted in peak deterioration. Removing protein through denaturation and precipitation is a highly effective method for biological sample pretreatment and is commonly used for plasma and blood samples before analysis. Several precipitation reagents, including TCA (10% *w*/*v*), perchloric acid (PCA, 6% *w*/*v*), acetonitrile, acetone, and methanol, were evaluated. Significant interference peaks around the FTS elution time were found after precipitation by acetonitrile, acetone, and methanol. The addition of PCA resulted in the instability of FTS after 4 h at room temperature. Deproteinization using TCA was selected, which led to a region with minimal interference from 10 to 14 min (Figure 2A) and provided an acceptable recovery for FTS (approximately 80%). Regarding detection and separation, we used a mobile phase composed of acetonitrile and phosphate solution (adjusted to pH 2.5 with phosphoric acid) to give a retention time of 13.1 min for FTS. The developed HPLC-EC method has a 10-fold improvement in sensitivity compare with previously used HPLC-UV methods (LOQ 0.2 μg/mL) [1,12].

The active compound releasing from hydrogel was affected by several factors such as the compound’s partition between water and polymer, the water diffusion rate into the polymer, the drug diffusion rate from the hydrogel, and the hydrogel dissolution under our experimental conditions. To determine the drug release profile, the accumulative release ratio of FTS in hydrogel formulations (F2, F3 and F5) was fitted to various mathematical models, namely a zero-order kinetic model, a first-order kinetic model, and a Higuchi model. The coefficient of determination (*r^2^*) of the three formulations is listed in Table S2. The results of the mathematical models showed that the three formulations were best fitted by the Higuchi model (*r^2^* = 0.9179, 0.9114, and 0.9223 for F2, F3, and F5, respectively), indicating that the drug release followed Fickian diffusion [28]. The pharmacokinetic studies in rats demonstrated a significant (*p* < 0.05) difference in AUC (2.1 times) and mean residual time (5.5 times) between subcutaneous administration of FTS-loaded hydrogel and subcutaneous administration of FTS solutions. Studies have reported t_1/2_ values of FTS after intravenously administration in rats around 73–77 min [2]. In this study, the t_1/2_ was prolonged from 76.6 to 516.6 min after the subcutaneous administration of FTS-loaded hydrogel, which confirms the extended effect of the developed formulation.

## 4. Experimental Section

### 4.1. Chemicals and Reagents

Forsythiaside (purity > 98%) was obtained from Fusol Material Ltd. (Tainan, Taiwan). Pinoresinol (purity > 98%) was acquired as internal standard (ISTD) from Seedchem (Melbourne, Australia). Puronic F127, F68, and PEG 6000 were purchased from Sigma-Aldrich (St. Louis, MO, USA). Methanol and acetonitrile (HPLC grade) were supplied by Tedia (Fairfield, OH, USA). Water was purified using the Milli-Q system (Millipore, Bedford, MA, USA). HPLC grade solvents were filtered through a 0.45-μm membrane filter (Millipore) and degassed in an ultrasonic bath (Branson Model 3210, Danbury, CT, USA) before use.

### 4.2. Animals

Male Sprague-Dawley (SD) rats (200 ± 20 g) were purchased from BioLasco (Taipei, Taiwan). The animal experiment and caring protocol was reviewed and approved by the Institutional Animal Care and Use Committee of Kaohsiung Medical University (Approval number 102074). Animals were housed in standard laboratory conditions (temperature 25 °C ± 2 °C, relative humidity 50% ± 20%). Blood samples were collected at predetermined time points through jugular vein catheterization [29]. In brief, the rat was surgically implanted with a polyethylene tube (PE-50) in the right jugular vein. The PE-50 catheter was exteriorized, capped, and fixed in the dorsal neck region. The tubing patency was conserved by flushing with a normal saline solution (0.9% NaCl, *w*/*v*) containing heparin sodium (15 IU/mL).

### 4.3. HPLC-EC Conditions

Chromatographic analysis was conducted on a Chromaster HPLC system (Hitachi Ltd, Tokyo, Japan) consisting of a 5160 pump and a 5260 autosampler. FTS and IS were separated on a Kinetex XB-C18 column (100 mm × 2.1 mm, i.d. 2.6 μm, Phenomenex, Torrance, CA, USA). The mobile phase comprised an acetonitrile—0.05-M phosphoric solution (11.8:88.2, *v*/*v*), and the pH was adjusted to 2.5 by using *O*-phosphoric acid. The mobile phase was delivered at 400 μL/min with an injection volume of 20 μL. The LC-4C electrochemical detector (Bioanalytical Systems, Inc. (BAS), West Lafayette, IN, USA) consists of a cross-flow cell equipped with a glassy carbon electrode (GCE, dia. 3 mm × 2 electrodes, dual type, ALS Co. Ltd., Tokyo, Japan), a Ag/AgCl reference electrode (RE-6 model, BAS), and a MF-1044 type gasket. A potential of +900 mV relative to the reference electrode was applied to the GCE by using a filter setting of 0.1 Hz and a range of 20 nA. Chromatographic results were acquired and processed using the Chromaster System Manager software (Tokyo, Japan).

### 4.4. Preparation of Stock Solutions, Calibration Curves, and Quality Control Samples

FTS and ISTD were individually dissolved in methanol to yield stock solutions at 100 μg/mL for each compound. The stock solutions were stored at −70 °C. The FTS stock solution was serially diluted with 50% (*v*/*v*) methanol to produce working standard solutions. A seven-point calibration curve was constructed by spiking 20 μL of the working standard solution into 80 μL blank plasma to obtain final concentrations of 20, 25, 50, 100, 200, 500, and 1000 ng/mL. Samples of LQC, MQC and HQC were prepared in the same manner.

### 4.5. Plasma Sample Pretreatment

Ten percent (*w*/*v*) trichloroacetic acid (TCA) was added to the plasma sample for deproteinization. The ISTD (20 μL) was fortified into the plasma sample (100 μL) in a 1.5-mL plastic centrifuge vial, following which the TCA solution (40 μL) was added and vortexed for 1 min. The supernatant was kept after centrifugation (14,000× *g*, 10 min, 4 °C) and analyzed through HPLC-EC.

### 4.6. Method Validation

#### 4.6.1. Selectivity

Blank plasma samples from six different rats were prepared according to the aforementioned process. The selectivity was checked by inspecting interference peaks appearing around the retention times of FTS and ISTD.

#### 4.6.2. Linearity and Sensitivity

The calibration curve was derived through least-square linear regression of the peak-area ratio versus FTS concentrations. Linearity was defined a coefficient of determination (*r*^2^) exceeding 0.995. The LOQ was defined as the lowest concentration of the calibration curve with acceptable accuracy and precision. The LOD was defined as the concentration at a signal-to-noise ratio of 3:1 [29].

#### 4.6.3. Recovery

The recovery of FTS after protein precipitation was calculated by comparing the peak areas of spiked samples to working standard samples with corresponding concentrations at 40, 400, and 750 ng/mL (*n* = 3). The recovery of ISTD was evaluated similarly.

#### 4.6.4. Accuracy and Precision

LQC, MQC, and HQC samples were analyzed within 1 and 6 days, respectively, to evaluate intra- and interday accuracy and precision of the developed HPLC-EC method. Accuracy (%) was expressed by dividing the measured concentration by the nominal concentration, and precision (%) was expressed as the relative standard deviation (RSD).

#### 4.6.5. Stability

Sample stability in the autosampler during HPLC analysis was evaluated at ambient temperature and at three concentrations (LQC, MQC and HQC) of samples analyzed after 0, 16, and 24 h. The storage stability was evaluated by determining whether the stock solutions remained stable at −70 °C. Stock solutions at 0, 1, 2, and 3 months were diluted with 50% (*v*/*v*) methanol to 400 ng/mL and analyzed through HPLC. The stability of benchtop and freeze/thaw for 3 cycles were also evaluated at three different concentrations (LQC, MQC and HQC).

### 4.7. Preparation and of Physicochemical Evaluation of Hydrogel Formulations

The procedures were established according to the cold method [30]. FTS was dispersed in different formulations containing F127 alone or with the addition of F68 or PEG 6000 at 4 °C ± 2 °C under magnetic stirring. Each formulation was equilibrated in a 4 °C refrigerator until turning into a clear solution. The formulations are listed in Table 5. The T_sol-gel_, viscosity, and gelling time of each formulation were measured using a DV2TLV viscometer (Brookfield, MA, USA) with a SC4-31 spindle, thermostatically controlled using a B401H circulating water bath. Formulations were examined in the 10 °C–46 °C range in increments of 2 °C. After the set temperature was reached, a 5-min quiet time was observed before the viscosity test. When the viscosity increased 1000-fold, the temperature was recorded and defined as T_sol-gel_. The gelling time was defined as the time required to reach 50,000 cps at T_sol-gel_. The water bath was set at 37 °C ± 0.5 °C, and cold hydrogel (4 °C) was tested.

The membrane-less method was used for hydrogel dissolution [24,31]. Each formulation (4.0 mL) was transferred into a conical glass test tube and was incubated for 30 min in a shaking water bath (37 °C, 70 rpm) to solidify the hydrogel. An aliquot of 5 mL in phosphate buffered saline (1.0 M, pH 7.4, 37 °C) was added gently without disturbing the surface of the solidified gel. The dissolution medium was removed at predetermined time points, and the weight of the residual gel and test tube was measured. The residual gel in the test tube was refilled with fresh dissolution medium (5.0 mL) to continue dissolution. The loss of weight was calculated as the difference between the empty conical glass test tube and the tube with hydrogel to estimate the dissolution rate. The procedures for evaluating FTS release from F2, F3 and F5 were performed according to a previous report [24]. Dissolution samples were analyzed according to a previous method with appropriate modifications [13]. The data obtained from in vitro drug release studies of F2, F3, and F5 were further fitted to various mathematical models, including zero-order kinetic, first-order kinetic, and Higuchi model, to evaluate the drug release kinetics [28].

### 4.8. Pharmacokinetic Evaluation

Twelve SD rats were randomly divided into three groups (*n* = 4 in each group). The first group received 3 mg/kg, IV FTS dissolved in 0.9% NaCl through femoral vein catheterization. The second group received the same dose of FTS solution (3 mg/kg) through subcutaneous (SC) injection in the dorsal surface of the cervical region. The third group received the optimal hydrogel formulation (3 mg/kg) through subcutaneous (SC) injection. Blood samples were taken at predetermined time points, and plasma fractions were collected and stored at −70 °C until analysis.

### 4.9. Data Analysis

Data were presented as means ± standard deviation (STD). A standard noncompartmental pharmacokinetic model was used to calculate the following parameters [32,33]. The area under the curve (AUC) of plasma concentration versus time from zero to the last time point was calculated using the trapezoidal method. The mean retention time (MRT) was estimated as AUMC/AUC, where AUMC is the area under the first-moment curve. Clearance was calculated as dose/AUC, and the volume of distribution (V_ss_) was Cl × MRT. Finally, half-life (t_1/2_) was determined at ln 2/K_e_. Comparison of the data was assessed through one-way analysis of variance and followed by Fisher’s least significant difference by using the SPSS software (IBM SPSS Statistics 14.0). *p* < 0.05 was considered significant.

## 5. Conclusions

A sensitive and validated HPLC-EC method was developed for quantifying FTS in rat plasma. The validation results indicated that this method has good linearity from 20 to 1000 ng/mL, with *r*^2^ > 0.999. An efficient deproteination method using TCA achieved FTS recovery exceeding 79%. Precision and accuracy varied by less than 10%, indicating the reliability and repeatability of the method. The method was applied to the in vivo pharmacokinetic evaluation of FTS-TIGS. The pharmacokinetic results showed a significant extension of the FTS residual time using our hydrogel formulation as evidenced by the significantly increased AUC and t_1/2_, as well as the reduced Cl of FTS.

## Figures and Tables

**Table 1:** Accuracy and precision of QC samples for FTS analysis.

Nominal Concentration (ng/mL)	Observed Concentration (ng/mL)	Precision (%)	Accuracy (%)
Intra-day			
20	18.2 ± 1.2	6.6	91.2 ± 6.0
40	42.6 ± 2.8	6.6	106.5 ± 7.0
400	378.0 ± 28.3	7.5	94.5 ± 7.1
750	761.2 ± 18.4	2.4	101.5 ± 2.5
Inter-day			
20	17.9 ± 1.3	7.6	89.3 ± 6.7
40	38.6 ± 3.5	9.0	96.6 ± 8.7
400	389.6 ± 21.7	5.6	97.4 ± 5.4
750	748.2 ± 7.8	1.0	99.8 ± 1.0

Data was shown as mean ± standard deviation (*n* = 6).

**Table 2:** Stability of QC samples of FTS and ISTD in autosampler.

Concentration (ng/mL)	Time (h)		
	0	16	24
40	100	103.8 ± 1.1	102.4 ± 9.0
400	100	104.8 ± 1.8	105.4 ± 7.3
750	100	106.4 ± 1.3	107.1 ± 2.7
2000 (ISTD)	100	95.0 ± 1.9	96.3 ± 4.4

Data was shown as percentage of peak area compared to the initial area as mean ± standard deviation (*n* = 3).

**Table 3:** Stability of FTS after freeze/thaw and benchtop storage conditions.

Stability	LQC (40 ng/mL)		MQC (400 ng/mL)		HQC (750 ng/mL)	
	Precision (%)	Accuracy (%)	Precision (%)	Accuracy (%)	Precision (%)	Accuracy (%)
Benchtop for 8 h	2.7	96.8 ± 2.7	6.3	99.4 ± 6.3	2.9	100.0 ± 2.9
Freeze/thaw for 3 cycles	6.7	96.1 ± 6.4	2.4	92.0 ± 2.2	5.3	106.1 ± 5.6

Data expressed as mean ± standard deviation (*n* = 3).

**Table 4:** Dilution integrity test for FTS analysis.

Concentration in Spiked Rat Plasma (ng/mL)	Diluted Times	Nominal Concentration (ng/mL)	Precision (%)	Accuracy (%)
1500	2	750	3.2	97.7 ± 3.1
40,000	50	800	4.2	95.0 ± 4.0

Data was shown as mean ± standard deviation (*n* = 5).

**Table 5:** Formulations, T_sol-gel_ and gelation time of FTS thermo-sensitive hydrogel.

Formulation	Composition			Viscosity at 26 °C (cp)	T_sol-gel_ (°C)	Average Gelling Time (min)	Maximum Viscosity (cp)	Complete Dissolution Time (h)	Total Score
	F127 (%)	F68 (%)	PEG 6000 (%)						
F1	17	-	-	1,266,000	26	1.1	2 × 10^6^	120	N/A
F2	17	1	-	138	28	1.4	2 × 10^6^	96	15
F3	17	2	-	114	34	3.0	2 × 10^6^	96	13
F4	17	3	-	102	36	4.5	2 × 10^6^	72	6
F5	17	-	0.5	400	28	1.7	1.86 × 10^6^	72	14
F6	17	-	1	120	30	1.6	1.33 × 10^6^	72	12
F7	17	-	1.5	120	38	5.2	7.2 × 10^5^	48	1

N/A: Not applicable due to high viscosity at 26 °C.

**Table 6:** Pharmacokinetic parameters of FTS.

Parameter	Unit	Group		
		1 (Solution, IV)	2 (Solution, SC)	3 (F5-hydrogel, SC)
C_0_	ng/mL	36,385 ± 10,387	N/A	N/A
C_max_	ng/mL	N/A	1518 ± 162	498 ± 145 *
t_1/2_	min	57.9 ± 17.0	76.6 ± 7.2	516.6 ± 111.9 *
AUC_0→t_	min ng/mL	149,462 ± 32,852	74,894 ± 18,372	161,396 ± 21,771 *
AUC_0→__∞_	min ng/mL	155,726 ± 29,172	80,321 ± 16,671	184,856 ± 28,599 *
Cl	mL kg/min	19.9 ± 4.6	38.5 ± 7.6	16.5 ± 2.3 *
V_d_	mL/kg	1683.6 ± 669.6	4261.0 ± 953.8	12,292.1 ± 3333.1 *
MRT	min	20.0 ± 6.0	75.5 ± 7.8	417.1 ± 57.0 *

N/A: not applicable. C_0_/C_max_: peak plasma concentration of FTS, t_1/2_: half-life, AUC_0→t_: Area under the plasma concentration-time curve from zero (0) hours to time (t), AUC_0→__∞_: Area under the plasma concentration-time curve from zero (0) hours to infinity (∞), Cl: clearance, V_d_: volume of distribution, MRT: mean residence time. Data was expressed with mean ± standard deviation (*n* = 4). * *p* < 0.05 compared with group 2 (Solution, SC).

**Table 7:** Comparison of HPLC-based analytical method performance for forsythiaside.

Detection Method	Sample Type	Limit of Quantification (ng/mL)	Linear Range (ng/mL)	Run Time (min)	Reference
UV	Canine plasma	52	52–13,330	18	[9]
UV	Canine plasma	78	78–40,000	30	[10]
UV	Rat plasma	67	67–26,667	20	[13]
MS/MS	Rat plasma	2.0	2.0–50.0 and 50.0–5000.0	10	[2]
MS/MS	Rat plasma	2	2–500	29	[14]
MS/MS	Rat plasma	5.15	5.15–5150	11	[15]
UPLC–MS/MS	Rat plasma	0.46	0.46–236.10	8	[16]
UPLC–MS/MS	Rat plasma	0.4795	0.4795–982.1	5.5	[17]
MS	Rat plasma	1.294	1.294–2587.5	10	[26]
EC	Rat plasma	20	20–1000	30	This study

UV: ultraviolet detection. MS/MS: tandem mass spectrometric detection. MS: mass spectrometric detection. UPLC–MS/MS: ultra-performance liquid chromatography with tandem mass spectrometric detection. EC: electrochemical detection.

## Supplementary Materials

The following are available online at http://www.mdpi.com/1420-3049/21/10/1384/s1.
