# Supplementary material for: Bioanalytical Method Development Using Liquid Chromatography with Amperometric Detection for the Pharmacokinetic Evaluation of Forsythiaside in Rats
Source: Molecules. 2016 Oct 16;21(10):1384. doi: 10.3390/molecules21101384 (PMC6274433; doi:10.3390/molecules21101384)
Supplement: Supplementary file 1 [file molecules-21-01384-s001.pdf]

Supplementary Materials: Bioanalytical Method Development Using Liquid Chromatography with Amperometric Detection for the Pharmacokinetic Evaluation of Forsythiaside in Rats

Yu-Tse Wu, Meng-Ting Cai, Chih-Wei Chang, Ching-Chi Yen and Mei-Chich Hsu

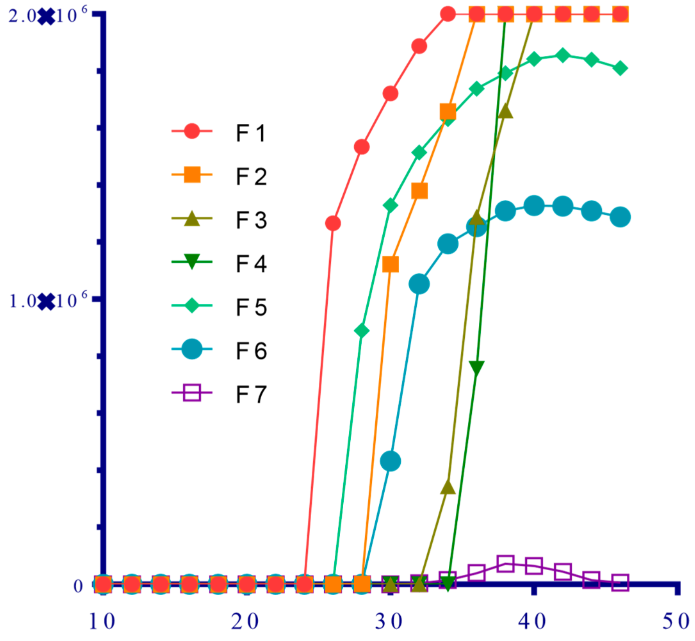

Figure S1. Figure S1. The temperature-viscosity profile of formulations 1–7.

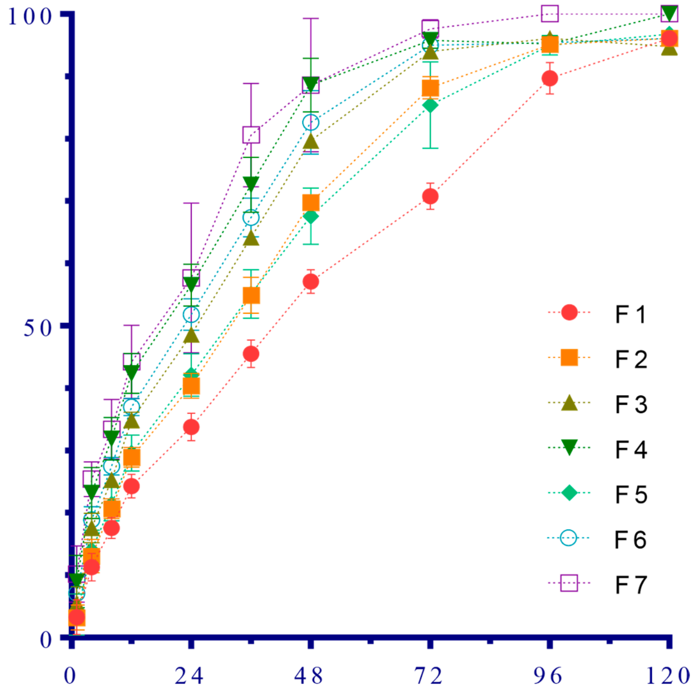

Figure S2. Gel dissolution of formulations 1–7.

Table S1. Scoring criteria for hydrogel formulations.

Property	Range	Score
Tsol-gel (°C)	32–34	6
	28~30	4
	<28 or >34	0
Gelation time (sec)	<120	4
	120~150	2
	>150	0
Maximum viscosity (cps)	>1.5 × 10^6	4
	>1 × 10^6	2
	<1.0 × 10^6	0
Complete dissolution time (h)	96	3
	72	2
	48	1

Table S2. Release model fitting of FTS-loaded hydrogel formulations.

Model	*r* of Formulations		
	F2	F3	F5
Zero-order kinetic	0.727	0.7153	0.7351
First-order kinetic	0.4237	0.4066	0.4422
Higuchi	0.9179	0.9114	0.9223
